# Night Eating Syndrome and Its Association With Depression Among College Students: A Cross-Sectional Study

**DOI:** 10.7759/cureus.76109

**Published:** 2024-12-20

**Authors:** Geethanjali Murthy, Pavan Kumar Gollapalli, M. Jasmine, B.N. Surya, Manoj P, Nishanth Kumaran Shri P M, Rehana Syed, Vijayalakshmi S, Kesavan Sundaraboopathy, Anand Kumar

**Affiliations:** 1 Community Medicine, Chettinad Hospital and Research Institute, Chengalpattu, IND; 2 Department of Community Medicine, Chettinad Hospital and Research Institute, Chengalpattu, IND; 3 Community Medicine, Chettinad Hospital and Research Institute, Chettinad Academy of Research and Education, Chengalpattu, IND; 4 Preventive Medicine, Chettinad Hospital and Research Institute, Chengalpattu, IND; 5 Community and Family Medicine, Chettinad Hospital and Research Institute, Chengalpattu, IND; 6 Community and Family, Chettinad Hospital and Research Institute, Chengalpattu, IND

**Keywords:** body mass index, college students, mental health, night eating syndrome, young adult

## Abstract

Introduction

Night eating syndrome (NES) is categorized as an eating disorder characterized by a delayed circadian rhythm of food intake and involves evening hyperphagia and/or nighttime awakening and food consumption two or more times per week. Young adults showed a higher prevalence of night eating, and students who reported high stress, irregular sleep patterns, and disordered eating may be more likely to develop NES symptoms.

Objective

This study aimed to assess the prevalence of Night Eating Syndrome among college students in Chengalpattu district of Tamil Nadu (India) and to find out the association between NES and socio-demographic variables and depression among the students.

Methodology

A cross-sectional study was conducted among 223 college students in the Chengalpattu district. They were selected through a simple random sampling method. The Revised Night Eating Diagnostic Questionnaire was used to assess NES, and the Patient Health Questionnaire-9 (PHQ-9) was used to assess the level of depression among students.

Results

The prevalence of NES among college students was found to be 37.3%. Out of 223 students, 38 had mild, 26 had moderate, and 19 had severe NES. We also found that depression (<0.001) and BMI (<0.001) were statistically associated with NES in the study population.

Conclusion

Night Eating Syndrome is a significant concern among college students. It is characterized by a delayed pattern of food intake, often leading to disrupted sleep and eating cycles, and is frequently accompanied by depression. The prevalence of NES in this demographic highlights the need for increased awareness and targeted interventions.

## Introduction

Despite being first described in scientific journals over half a century ago, night eating syndrome (NES) is a unique eating problem that receives little recognition or clinical treatment. In the 1950s, NES was initially identified in a subgroup of obese people undergoing weight-loss therapy. According to the study done by Stunkard et al, the NES subgroup had early sleeplessness or difficulty going asleep, evening hyperphagia, and minimal morning hunger [1]. Besides this first description, different criteria have been utilized to diagnose NES, with Birketvedt et al introducing nocturnal ingestions - that is, awakening during the sleep period to eat - as a significant alteration [2].

Research has shown correlations between NES and eating disorders (ED), maladaptive coping, and poor psychological and physical well-being [3]. In a research study, young adults were more likely than the rest of the population to experience nighttime hyperphagia [4]. Estimates of the exact amount of NES in the overall population vary from 1.5% to 4.6%, making it difficult to pinpoint [5]. This prevalence is significantly higher among people with psychiatric problems, especially depression (up to 15%), and obesity (3% to 15%) [6].

Approximately 300 million individuals worldwide, or 4.4% of the total population, suffer from depression, making it one of the most prevalent mental health conditions. The World Health Organization lists depression as the third leading cause of illness globally [7]. Researchers have made numerous attempts to pinpoint dietary components associated with depression. It has been observed that NES sufferers experience more severe depressive symptoms [8].

It was also observed that these symptoms appeared to get worse under stress and might get better if the person was taken out of the stressful situation. While there has been frequent reporting of a link between NES and depression, stress wasn't included as any of the diagnostic criteria mentioned above [9].

The prevalence of NES among Indian students has only been studied once, and the results show that it is at 54% [10]. Numerous student populations' NES has been linked through research to mental health issues, especially depression. Still, not enough research has been done on NES, especially in the unique setting of college students, despite its importance to global health and its ability to affect people's quality of life. Hence, this study aims to evaluate the prevalence of NES among college students in the Chengalpattu district of Tamil Nadu, India, and examine its association with socio-demographic variables and depression among the students.

## Materials and methods

Between July and September 2024, a community-based cross-sectional survey was conducted among the colleges in Tamil Nadu's Chengalpattu district. The study comprised male and female engineering college students who were older than eighteen. College students with a history of recent bereavement experiences or those with a diagnosis of neuropsychiatric disorder were not included. The Institutional Human Ethics Committee for Students Research of Chettinad Academy of Research and Education (CARE) (CARE IHEC -I) approved the study proposal (Ref No.: IHEC-I/301/24).

Based on the study by Kaur M et al [10], the sample size was determined to be 203, taking into account a 54% prevalence of night eating syndrome, a 95% confidence interval, and a 7% acceptable error. Interviews were conducted with 223 college students in order to account for a 10% non-response rate. Design effect was not taken into consideration as simple random sampling was used.

One block was chosen using a simple random selection method out of eight blocks in our hospital's field practice area. Three engineering colleges out of eight were selected using the simple random sample method, and a list of qualified students was created based on the inclusion and exclusion criteria. The study participants were selected from the line list using randomly generated computer numbers.

Data was gathered using a pre-tested, three-section, semi-structured questionnaire (see Appendices). Sociodemographic information was included in the first section, and the study population's level of NES was evaluated using the redesigned Night Eating Diagnostic Questionnaire (NEDQ), a 21-item survey with both closed-ended and open-ended items (with sub-questions) [11]. The final scoring was determined by combining the six criteria - A, B, C, D, E, and F (A. One or both of the following 1) At least 25% of food intake is consumed after the evening meal. 2) At least two nocturnal eating episodes per week; B. Awareness and recall of evening and nocturnal eating episodes are present; C. At least three of the following. 1) Lack of desire to eat in the morning and/or breakfast is omitted four or more times per week. 2) Presence of a strong urge to eat between dinner and sleep onset and/or during the night. 3) Sleep maintenance and/or onset insomnia are present four or more times per week. 4) Presence of a belief that one must eat in order to initiate or return to sleep. 5) Mood is frequently depressed or mood is worse in the evening; D. The disorder is associated with significant distress and/or impairment in functioning; E. Maintenance of disordered eating for at least 3 months; F. The disorder is not secondary to substance abuse or dependence, medical disorder, medication, or another psychiatric disorder.) that were developed in accordance with Geliebter et al. [11]. A patient health questionnaire (PHQ-9) was used in the third section to gauge the degree of depression [12]. The severity of depression was measured using a 4-point Likert scale with nine items. Scores ranged from 0 (not at all) to 3 (almost daily) for each question. The ultimate score was calculated by adding up all of the responses.

Depression was categorized into five groups based on the final score: minimal depression was represented by a score of 1 to 4, mild depression by a score of 5 to 9, moderate depression by a score of 10 to 14, moderately severe depression by a score of 15 to 19, and severe depression by a score of 20 to 27. After gaining the participants' informed consent, data was gathered. A Microsoft Excel (Microsoft Corporation, Redmond, USA) spreadsheet and the Statistical Package for Social Sciences version 21 (IBM Corp., Armonk, USA) were used for data entry and analysis. While frequency and percentage were used to describe qualitative factors, mean and standard deviation were used to describe quantitative variables. Chi-Square test was used to find the association between NES and its associating factors and depression. P < 0.05 was considered significant.

## Results

The mean age of the participants was 20.03 ± 1.56 years. Almost 68% of the participants were females. The body mass index of the students was normal for 65.9%; only 14.8% and 4.5% were found to be overweight and obese (Table 1). The study population was classified as underweight if their BMI was less than 18.5; normal if their BMI was between 18.5 and 22.9; overweight if their BMI was between 23 and 24.9; obese class 1 if their BMI was between 25 and 29.9; and obese clas 2 if their BMI was greater than 30 (Table 1).

**Table 1:** Demographic details of the study participants *BMI according to WHO Asian classification

Variable		No of participants (%) (n=223)
Age		20.03 ± 1.56
Gender	Male	71 (31.8)
	Female	152 (68.2)
Religion	Hindu	162 (72.7)
	Christian	27 (12.1)
	Muslim	34 (15.2)
Family	Nuclear	157 (70.4)
	Joint	53 (23.7)
	Extended	13 (5.9)
Body Mass Index (BMI)*	Underweight (<18.5 kg/m^2^)	33 (14.8)
	Normal (18.5-22.9 kg/m^2^)	147 (65.9)
	Overweight (23-24.9 kg/m^2^)	33 (14.8)
	Obese class 1 (25-29.9 kg/m^2^)	10 (4.5)
	Obese class 2 (>30 kg/m^2^)	0

**Figure 1:**
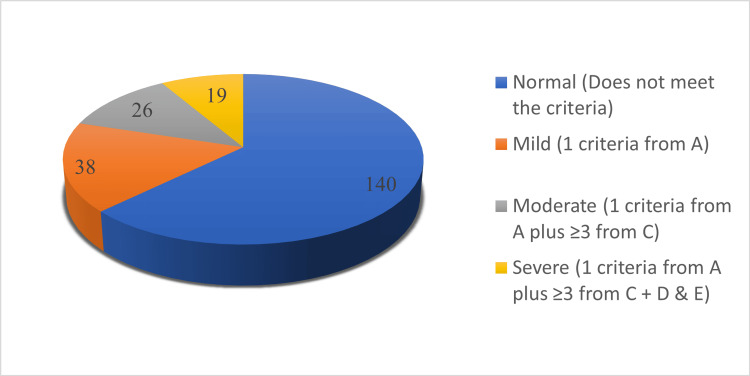
Distribution of night eating syndrome among the study participants (n=223) A. One or both of the following 1) At least 25% of food intake is consumed after the evening meal. 2) At least two nocturnal eating episodes per week; B. Awareness and recall of evening and nocturnal eating episodes are present; C. At least three of the following. 1) Lack of desire to eat in the morning and/or breakfast is omitted four or more times per week. 2) Presence of a strong urge to eat between dinner and sleep onset and/or during the night. 3) Sleep maintenance and/or onset insomnia are present four or more times per week. 4) Presence of a belief that one must eat in order to initiate or return to sleep. 5) Mood is frequently depressed or mood is worse in evening; D. The disorder is associated with significant distress and/or impairment in functioning; E. Maintenance of disordered eating for at least 3 months; F. The disorder is not secondary to substance abuse or dependence, medical disorder, medication, or another psychiatric disorder. (Based on Geliebter et al. [11])

Of the reported NES, 38 (17%) had mild NES, 26 (12%) had moderate NES, and 19 (8%) had severe NES (Figure 1).

Table 2 depicts the prevalence of depression among the study participants - 66.4% reported having minimal or no depression and 33.6% had some form of depression.

**Table 2:** Level of depression with Patient Health Questionnaire (PHQ-9)

Severity	Score range	No of participants (%) (n=223)
Normal / Minimal	1 – 4	148 (66.4)
Mild	5 – 9	31 (13.9)
Moderate	10 – 14	22 (9.9)
Moderately severe depression	15 – 19	17 (7.6)
Severe	20 – 27	5 (2.2)

The majority of those with depression were found to have NES, and the association was proven statistically (p<0.001). It was also found that those with depression had 2.5 times higher odds of having NES (Table 3).

**Table 3:** Association between night eating syndrome and the level of depression *Chi-square test, p<0.05 is significant

Depression	Night eating syndrome		p-value	Odds ratio	95% CI
	Absent	Present			
Normal	104	44	<0.001*	2.56	1.44 – 4.54
Depression	36	39			

The analysis identified several factors associated with the presence of NES among the participants. Gender is significantly associated, with females being twice as likely to have NES compared to males (OR = 2.01, 95% CI: 1.09-3.69). BMI shows a strong association with NES: obese individuals are significantly more likely to have NES (OR = 6.8, 95% CI: 2.33-19.85), followed by overweight individuals (OR = 3.53, 95% CI: 1.43-8.74). Family type and religion are not significantly associated with NES. This suggests that while gender and BMI are factors associated with NES, family structure and religious affiliation do not play a significant role in its prevalence (Table 4).

**Table 4:** Association between night eating syndrome and socio-demographic characteristics *p<0.05 is significant

Variables	Night eating syndrome		Total (n= 223)	Chi-square χ^2^	Unadjusted odd’s ratio (95% CI)	p-value
	Present n (%) n = 83 (37.3%)	Absent n (%) n = 140 (62.7%)				
Gender						
Female	67	85	152	5.148	2.01 (1.09-3.69)	0.023*
Male	20	51	71			
BMI						
Obese	15	5	20	21.867	6.8 (2.33 – 19.85)	0.005*
Overweight	14	9	23		3.53 (1.43 – 8.74)	0.008*
Underweight	9	24	33		0.85 (0.37 – 1.97)	0.705
Normal	45	102	147		1	1
Family						
Nuclear	59	98	157	2.147	0.51 (0.16 – 1.6)	0.254
Joint	17	36	53		0.40 (0.11 – 1.4)	0.151
Extended	7	6	13		1	1
Religion						
Christian	7	20	27	1.778	0.565 (0.226 – 1.412)	0.221
Muslim	14	20	34		1.129 (0.531 – 2.397)	0.752
Hindu	62	100	162		1	1

## Discussion

The disease is known as night eating syndrome, which is typified by sleeplessness, evening hyperphagia, and morning anorexia. The present study reported a prevalence of NES and depression of 37.3% and 33.6%, respectively, among the student population. College students were known to suffer from night eating syndrome frequently, and this group was also highly likely to experience depression. This study discovered that there is an association between NES and depression. The study found a positive correlation between NES and BMI, with individuals experiencing NES tending to have higher BMIs.

A similar study conducted in Punjab among students found a 54% prevalence of NES and 33% depression in their study population. The association between NES and depression was found to be statistically significant (p <0.001). The association between BMI and NES was also found to coincide with the present study findings (p<0.021) [10].

Similarly, a study by Zaidi et al in Karachi, Pakistan among college students found that 49.3% were suffering from NES and 27.8% had a prevalence of depression. Depression (p<0.03) and overweight (p <0.001) were found to be statistically significant with NES in this study [13].

The prevalence of NES among the student population (37.3%) in this study was found to be higher when compared to the general population (30%) [14]. Also, studies conducted in different regions of the world among student populations showed varying ranges of NES. Elsadek AM et al study reported a prevalence of 5.8% among Egyptian students [15]. Another study conducted in the US by Nolan LJ et al among college students found a 4.2% prevalence of NES [16]. Feng Guo et al conducted a similar study in China, which reported a 5.4% prevalence of NES and 21.3% of depression [17]. A 7.3% prevalence of NES was reported in a Saudi study by Sara Haneef et al [18] and a 9.5% prevalence of NES was reported in a Turkish study [19]. NES was found to be significantly associated with depression in both studies.

Factors like gender and body mass index were found associated with night eating syndrome in the present study. This association was addressed in various studies as psychologically females tend to cope with negative emotions through a mechanism of nighttime eating and also exhibit a higher level of cognitive restraint to maintain body weight leading to restricted daytime eating [20]. The present study's findings coincide with Amritwar et al’s where the majority of those with night eating syndrome were females (69.2%) and either overweight or obese (53.8%) [21]. Likewise, in a Karachi-based study [13] and various other studies [21,22], females tended to have NES when compared to males, and BMI was found to be positively correlated with NES.

The link between depressive symptoms and NES may be explained by the following mechanism. Data suggest that the pathogenesis of NES may involve a surge in serotonin transporter specific to the disease, which leads to an overall reduction in serotonin across the synapse. It is widely established that reduced circulating serotonin levels are connected with an increased risk of depressive symptoms [23].

Limitations

To the best of our knowledge, this is the first study done to report the NES prevalence and its association with depression in the Chengalpattu district. However, the limitation of this study is, first, there is little room for causal inference in cross-sectional studies. Second, selection bias could arise since study data were gathered from a single block. It is not possible to extrapolate the results to other age groups or geographic areas.

## Conclusions

These results highlight the significance of handling mental health concerns among students, especially those related to NES and depression, and especially in the early stages of life. Healthcare providers, educators, and other stakeholders can enhance the mental health and general well-being of this age group by offering focused support and resources to these students, including improved sleep hygiene, psychological interventions, mindfulness practices, and balanced eating. This emphasizes how important it is to take proactive steps to treat students' psychological health as a vital aspect of their overall health.

## Figures and Tables

**Figure 2:**
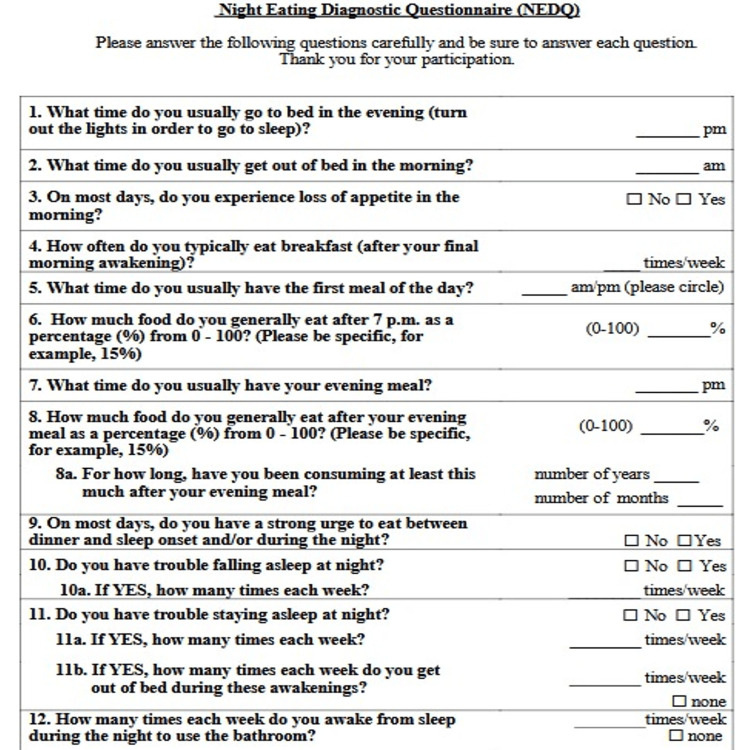
PART -B

**Figure 3:**
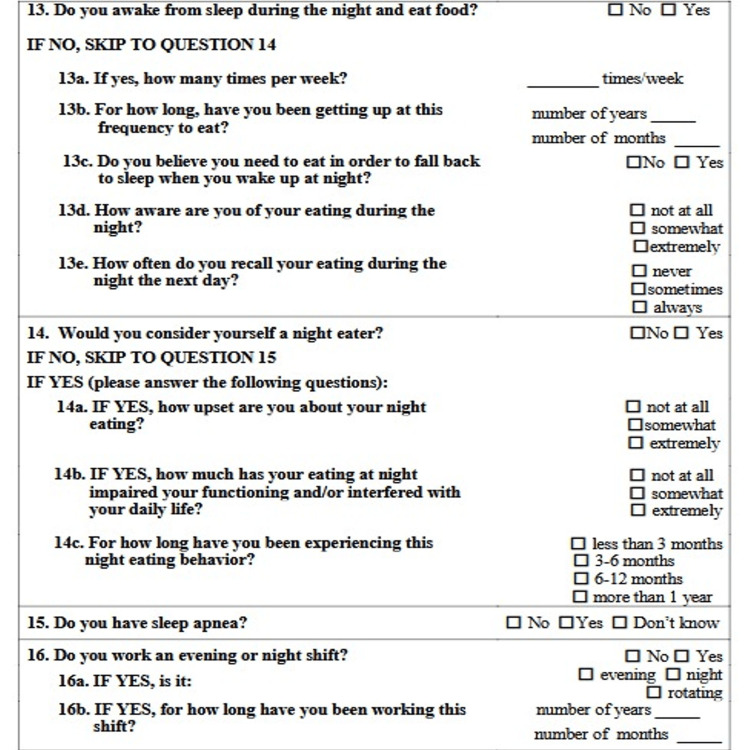
PART - B

**Figure 4:**
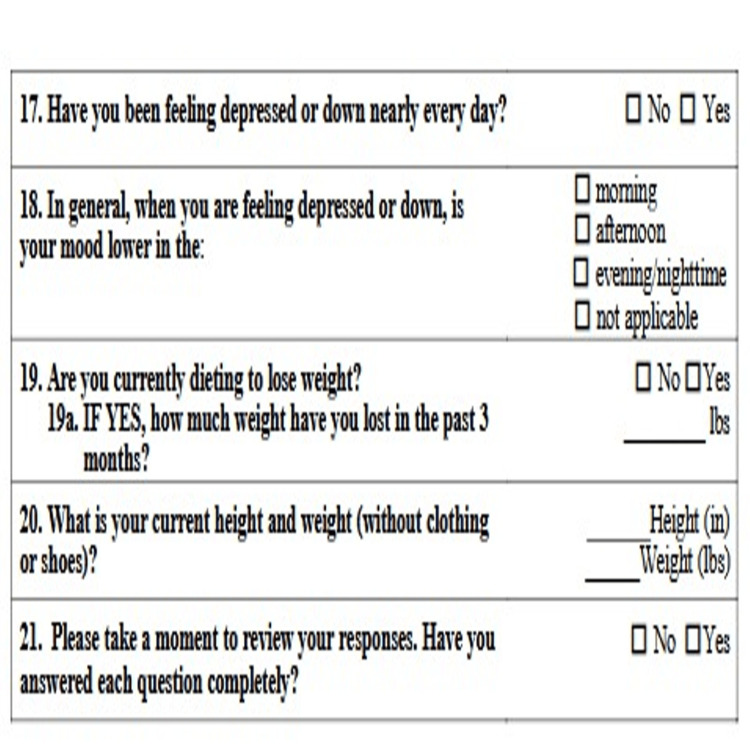
PART - B

**Figure 5:**
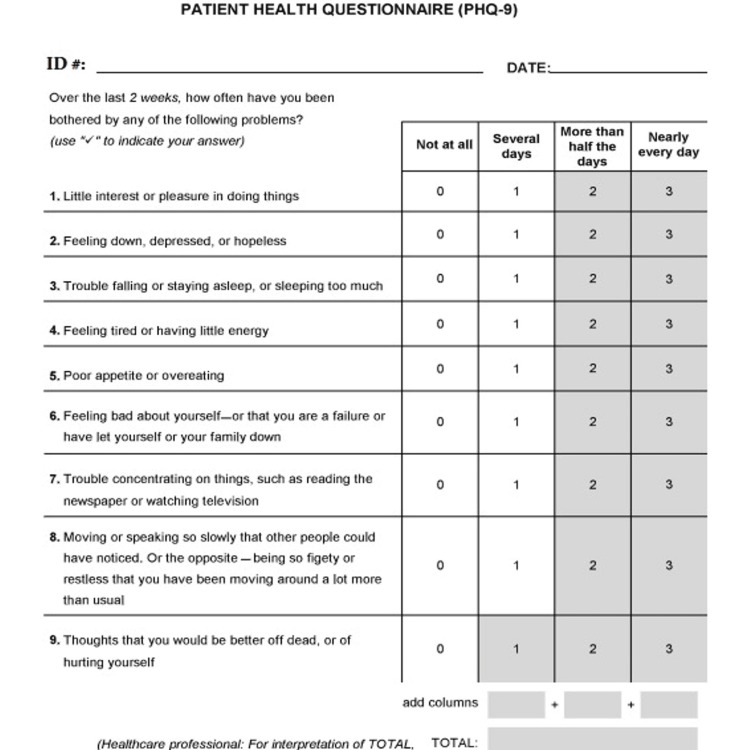
PART - C

## Appendices

QUESTIONNAIRE

PART - A

SOCIODEMOGRAPHIC PROFILE

1. NAME:

2. AGE:

3. GENDER: Male/ Female

4. ADDRESS:

5. EDUCATION:

- Course

- Year of Education

6. MONTHLY INCOME (FAMILY):

7. RELIGION: Hindu / Muslim /Christian /Others

8. TYPE OF FAMILY: joint / nuclear / Extended

9. NUMBER OF FAMILY MEMBERS

10. FATHER OCCUPATION

11. MOTHER OCCUPATION
